# Probiotic lactic acid bacteria alleviate pediatric IBD and remodel gut microbiota by modulating macrophage polarization and suppressing epithelial apoptosis

**DOI:** 10.3389/fmicb.2023.1168924

**Published:** 2023-06-15

**Authors:** Huiying Hua, Chun Pan, Xixi Chen, Mengxia Jing, Jinfang Xie, Yuanqi Gao, Jiebin Huang, Xuehua Chen, Yujing Gao, Chundi Xu, Pu Li

**Affiliations:** ^1^Department of Pediatrics, Ruijin Hospital, Shanghai Jiao Tong University School of Medicine, Shanghai, China; ^2^NHC Key Laboratory of Metabolic Cardiovascular Diseases Research, School of Basic Medical Sciences, Ningxia Medical University, Yinchuan, China

**Keywords:** pediatric IBD, macrophage polarization, intestinal epithelium apoptosis, *Akkermansia muciniphila*, *Pediococcus pentosaceus* CECT8330

## Abstract

**Introduction:**

The incidence of pediatric inflammatory bowel disease (PIBD) continues to rise. It was reported that the probiotic lactic acid bacteria *Pediococcus pentosaceus* (*P. pentosaceus*) can interfere with intestinal immunity, but it is still unknown whether it can alleviate PIBD and the concrete mechanism of immune regulation is unclear.

**Methods:**

For this study, 3-week-old juvenile mice were selected for modeling the development of PIBD. The mice treated with 2% DSS were randomly divided into two groups, which were given *P. pentosaceus* CECT8330 and equal amounts of solvent, respectively. The feces and intestinal tissue were collected for the mechanism exploration *in vivo*. THP-1 and NCM460 cells were used to investigate the effects of *P. pentosaceus* CECT8330 on macrophage polarization, epithelial cell apoptosis, and their crosstalk *in vitro*.

**Results:**

*P. pentosaceus* CECT8330 obviously alleviated colitis symptoms of juvenile mice, including weight loss, colon length shortening, spleen swelling, and intestinal barrier function. Mechanistically, *P. pentosaceus* CECT8330 could inhibit intestinal epithelial apoptosis by suppressing the NF-κB signaling pathway. Meanwhile, it reprogramed macrophages from a pro-inflammatory M1 phenotype to an anti-inflammatory M2 phenotype, leading to a decreased secretion of IL-1β which contributes to the reduction in ROS production and epithelial apoptosis. Additionally, the 16S rRNA sequence analysis revealed that *P. pentosaceus* CECT8330 could recover the balance of gut microbiota, and a significantly increased content of *Akkermansia muciniphila* was particularly observed.

**Conclusion:**

*P. pentosaceus* CECT8330 shifts macrophage polarization toward an anti-inflammatory M2 phenotype. The decreased production of IL-1β leads to a reduction in ROS, NF-κB activation, and apoptosis in the intestinal epithelium, all of which help to repair the intestinal barrier and adjust gut microbiota in juvenile colitis mice.

## Graphical Abstract

Schematic graph depicting how *P. pentosaceus* CECT8330 alleviates PIBD. 
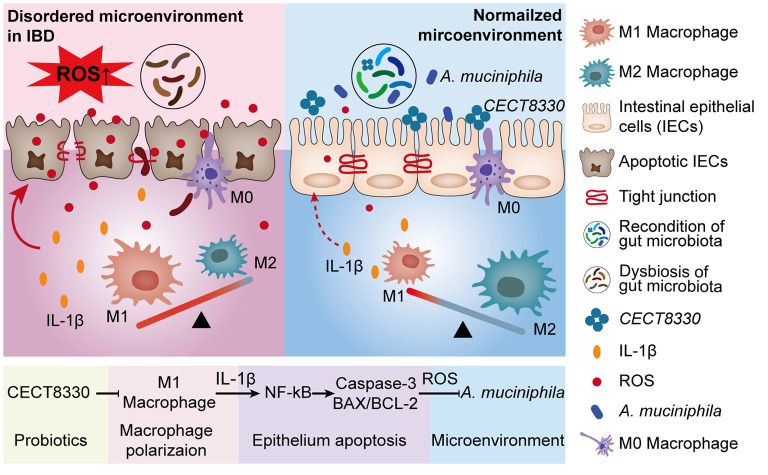

## 1. Introduction

Inflammatory bowel disease (IBD) is a chronic complex disease of children and adults comprising the chronic relapsing inflammatory disorders Crohn's disease (CD) and ulcerative colitis (UC) (Khor et al., 2011). The incidences of pediatric IBD (PIBD) were 23/100,000 person-years in Europe, 15.2/100,000 in North America, and 11.4/100.000 in Asia/the Middle East and Oceania (Sýkora et al., 2018). The steadily increasing incidence of PIBD over time indicates its emergence as a global disease (Kuenzig et al., 2022). Meanwhile, PIBD is different from adult IBD in terms of pathogenesis and treatment (Fuller, 2019). Moreover, the burden of IBD in children may be higher than in adults, and treatment requires a multidisciplinary approach aiming at eliminating symptoms, promoting growth and development, and supporting unrestricted living (Wilson and Russell, 2017). Therefore, it is necessary to develop more effective interventions and carry out special research on pediatric patients with IBD.

The etiology of PIBD is complex and is currently believed to be closely related to the composition, stability, and resilience of the intestinal microbiota (Mentella et al., 2020). Numerous studies have suggested that gut microbiota drive immune activation, and in turn, chronic inflammation can also influence the gut microbiome which leads to ecological dysregulation (Ni et al., 2017). In a cohort of 447 children with CD, it was found that the bacterial abundance of children with CD was significantly different from that of healthy children (Gevers et al., 2014). Clinical and experimental data suggest that gut dysbiosis plays a pivotal role in the pathogenesis of IBD (Nishida et al., 2018). In several IBD-related models, colitis symptoms either did not develop at all or were significantly attenuated under germ-free conditions, supporting the essential role of microbes in the development of IBD (Sartor, 2004). Hence, manipulating the microbiome becomes one promising approach for relieving intestinal inflammation and the treatment of PIBD.

Probiotics are living microbial food components, which can alter the microflora when ingested in adequate quantities, conferring a health benefit to the host (Howarth and Wang, 2013). Probiotics are beneficial to a faster recovery of disease, while the side effects are comparatively minimal (Kalakuntla et al., 2019). At present, reasonable amounts of probiotics are allowed to be used as dietary supplements in children and are safe in the treatment of pediatric intestinal diseases (Huang et al., 2022). Many reports provided evidence that some probiotic strains are useful for the treatment and prevention of PIBD (Jakubczyk et al., 2020). In children with different degrees of IBD, the clinical symptoms were significantly improved after probiotic compound preparation administration compared with the non-use group, and the recurrence rate was also reduced after remission (Henker et al., 2008; Huynh et al., 2009). Although the therapeutic effect of probiotics for PIBD is obvious, the exact function and mechanisms are unclear.

*Pediococcus pentosaceus* (*P. pentosaceus*), a member of the lactic acid bacteria, is a probiotic that has been shown to ameliorate inflammation (Bian et al., 2020). *P. pentosaceus* can ameliorate gut inflammation by not only maintaining gut epithelial integrity but also regulating immunity (Bian et al., 2020). One kind of *P. pentosaceus* named CECT8330 was used effectively in infantile crying syndrome clinically, due to its influences on the diversity and abundance of bacteria that correlate with a reduction in the total crying time (Tintore and Cune, 2017). It was reported that *P. pentosaceus* CECT8330 can induce IL-10 production, showing a broad-spectrum inhibitory activity against pathogens (Tintore et al., 2017). A recent study showed that probiotics *P. pentosaceus* CECT8330 had a protective effect by regulating the gut microbiota and the regulatory T cells on the colitis of 6-week-old mice (Dong et al., 2022). However, the immunologic regulation mechanism for *P. pentosaceus* CECT8330 changing gut microbiota and alleviating mice colitis remains unclear. Simultaneously, the 6-week-old mice belong to young adult mice (Zou et al., 2009; Ueno et al., 2018; Gorberg et al., 2021). Because intestinal and systemic immunity is still developing, there are obvious differences between juvenile and adult mice, just as there are also significant differences in the occurrence and development of IBD between children and adult patients. Therefore, the effect and mechanism of *P. pentosaceus* CECT8330 on colitis in juvenile mice and PIBD still lack more convincing research, which needs to be clarified.

In this study, 3-week-old juvenile mice (Caputi et al., 2017; Fuglsang et al., 2018) were given dextran sodium sulfate (DSS) to simulate the development of clinical PIBD. The effect of *P. pentosaceus* CECT8330 on disease development was evaluated, and the immunologic mechanism by which *P. pentosaceus* CECT8330 improves colitis was deeply investigated. Our study provides a concrete mechanism and scientific insight for better clinical treatment of PIBD.

## 2. Materials and methods

### 2.1. Chemicals and reagents

The *P. pentosaceus* CECT8330 was provided by MIHC Science Laboratory, DERBY CARE MEDICAL TECHNOLOGY CO., LIMITED (S.A, Spain). Dextran sodium sulfate (DSS) was purchased from Meilunbio (Dalian, China). RNAex Pro reagent and SYBR^®^ Green Premix Pro Taq HS qPCR Kit (Rox Plus) were purchased from Accurate Biology (Hunan, China). The kit of iScript™ cDNA synthesis was bought from Promega (WI, USA). Radio immunoprecipitation assay lysis buffer was obtained from Beyotime Biotechnology Co. Ltd (Nantong, China). The protease inhibitors, phosphatase inhibitors, SDS-polyacrylamide gel and secondary antibodies, and Super ECL Detection Reagent were acquired from Epizyme (Shanghai, China). Polyvinylidene difluoride membrane was purchased from Merck Millipore (MA, USA). The primary antibodies against ZO-1, OCCLUDIN, NF-κB P65, phospho-NF-κB P65, BAX, BCL-2, and IL-1β were bought from Cell Signaling Technology (MA, USA). The antibodies against glyceraldehyde-3-phosphate dehydrogenase (GAPDH) and β-Actin were obtained from Servicebio (Wuhan, China). The antibody against interleukin-6 (IL-6) was purchased from Affinity Biosciences (OH, USA). The antibody of Cleaved Caspase-3 and tissue ROS test kit (DHE) were bought from Absin Bioscience (Shanghai, China). The RPMI 1640 was obtained from Bioagrio (CA, USA). The fetal bovine serum was purchased from Tianhang Biotechnology Co., Ltd (Hangzhou, China), Phorbol-12-myristate-13-acetate (PMA) and ROS Assay Kit were bought from Beyotime Biotechnology Co. Ltd (Nantong, China). Lipopolysaccharide (LPS) from Escherichia coli O55:B5 was obtained from Sigma-Aldrich (MO, USA). Annexin V-FITC and PI were bought from Vayme (Nanjing, China). BIOG DNA Stool Kit was purchased from Baidai Biotechnology Co., Ltd (Changzhou, China). Amplex^®^ Red hydrogen peroxide/peroxidase detection kit was obtained from Thermo Fisher Scientific (MA, USA).

### 2.2. Animals

3-week-old male C57BL/6 mice were purchased from Phenotek Company (Shanghai, China), and all of them were group-housed at the Animal Research Center of Ruijin Hospital under specific pathogen-free conditions with a 12-h light–dark cycle and fed for 3 days after arrival.

Mice were randomly and equally divided into two groups: DSS and DSS+Pp (*n* = 6, each). Both of them were provided with 2% (wt/vol) DSS dissolved in drinking water for 7 days from the age of 4 weeks, while the DSS+Pp group was orally pre-administered by 200 μl of *P. pentosaceus* CECT8330 (1 × 10^9^ CFU per mouse daily) from the age of 3 weeks to the age of 5 weeks. The DSS group was given an equal volume of sunflower seed oil, which was the solvent of *P. pentosaceus* CECT8330. The fecal samples of mice were collected after the final challenge, and then, all mice were euthanized by carbon dioxide inhalation after fecal collection. The body weights of mice were recorded daily.

Colon tissue and blood were collected for further investigation. Distal parts of freshly isolated colon tissue were briefly washed with physiological saline and immediately fixed in 4% paraformaldehyde. Both the fecal samples and the rest part of the colon tissue were flash-frozen by liquid nitrogen and then stored at −80°C.

### 2.3. Histological analysis

Colon tissue was embedded in paraffin, and the sections of paraffin were stained with hematoxylin and eosin. For immunohistochemistry staining, the sections of colons were blocked with 5% goat serum and incubated with primary antibodies against ZO-1, OCCLUDIN, or P65 at 4 °C overnight after blocking endogenous peroxidase activity with 3% H_2_O_2_ and antigen retrieval with citric acid buffer. Then, the sections were incubated with secondary antibodies on the next day. The TdT-mediated dUTP nick-end labeling (TUNEL) assay was conducted following the manufacturer's protocols.

All the images above were scanned using Pannoramic MIDI and Pannoramic DESK (3DHIESTECH, Budapest, Hungary). Sections stained with H&E were randomly selected from each group for blind examination by an independent pathologist. As previously reported (Erben et al., 2014), the histopathological damages were assessed according to the histological activity index containing inflammatory cell infiltration and the intestinal architecture. All the other images were analyzed by ImageJ. The immunohistochemistry was quantitatively assessed by ImageJ with the IHC profiler plugin (https://sourceforge.net/projects/ihcprofiler), as described previously (Varghese et al., 2014). The expression levels of ZO-1 and OCCLUDIN were evaluated in a cytoplasmic staining pattern, while images of P65 and TUNEL were analyzed in a nucleus staining pattern by an IHC profiler. All positive zones were in the range of 0 to 180, while the intensity range of 236 to 255 was considered to have no contribution to the pathology score.

H_2_O_2_ was used to block the activity of endogenous peroxidase in immunofluorescent staining, followed by thermal antigen repair using Tris-EDTA. The sections of colons were blocked by 5% BSA. Sections were incubated with primary antibodies against CD68, CD86, and CD163 at 4°C overnight and secondary antibodies for 30 min at 37°C. Signal amplification was performed by tyramide conjugated to Cy3 or FITC. The fluorescence images were scanned using the TissueFAXS Plus automated acquisition system (TissueGnostics, Vienna, Austria). Analyze Particles tool in ImageJ was used to analyze the counts of macrophages in mice colons, as described previously (Ying et al., 2017).

### 2.4. ROS levels assay

Stimulated NCM460 cells were stained as previously described (Zou et al., 2017), and frozen colonic sections were incubated with DHE to evaluate the levels of ROS in mice colons according to the manufacturer's instructions. The fluorescence intensity of NCM460 cells was measured by an inverted fluorescence microscope at 488 nm excitation and 525 nm emission wavelength. The fluorescence intensity of colon tissue was measured at 531 nm excitation and 593 nm emission wavelength. The images were quantitatively assessed by ImageJ.

Amplex^®^ Red hydrogen peroxide/peroxidase detection kit was used to detect the extracellular ROS levels. The cell supernatants were collected after being stimulated, and the absorbance at wavelength 560 nm was detected by a fluorescent microplate reader.

### 2.5. Quantitative reverse transcription-polymerase chain reaction

Gene expression levels were quantitated by quantitative polymerase chain reaction (qPCR). The total RNA was extracted, and complementary DNA (cDNA) was synthesized according to the manufacturer's protocol. qPCR was performed with ABI QuantStudio6 (Applied Biosystems, MA, USA). The primer sequences are listed in Supplementary Table 1. The melting curve was used to confirm the specificity of the primers. The gene expression levels were normalized and calculated with the 2^−Δ*ΔCt*^ method for relative quantification normalized by the GAPDH gene expression.

### 2.6. Western blot

Colon tissue was homogenized using RIPA buffer which contained 1% protease inhibitor and 1% phosphatase inhibitor. Colonic tissue debris was removed by centrifugation at 12,000 rpm and 4°C for 15 min. An equivalent volume of samples was loaded and separated on 10% SDS-polyacrylamide gel electrophoresis and transferred onto PVDF membranes. The membranes were blocked with 5% non-fat milk at room temperature for 2 h and then incubated with primary antibodies overnight at 4°C. After incubating secondary antibodies for 1 h, the blot signals were detected by a chemiluminescent substrate. The densitometric analysis was performed by imageJ and all protein fractions were standardized according to GAPDH or β-Actin.

### 2.7. Luminex liquid suspension chip detection

Luminex liquid suspension chip detection was performed by Wayen Biotechnologies (Shanghai, China). The Bio-Plex Pro Human Chemokine Panel 40-plex kit was used following the manufacturer's instructions. In brief, the mice plasma was incubated in 96-well plates embedded with microbeads for 1 h and then incubated with detection antibody for 30 min. Subsequently, streptavidin-PE was added to each well for 10 min, and values were read using the Bio-Plex MAGPIX System (Bio-Rad, CA, USA).

### 2.8. 16S rRNA sequencing analysis

Total bacterial genomic DNA was extracted from fecal samples of mice by QIAamp DNA Stool Mini Kit. The quantity and quality of extracted DNAs were measured by NanoDrop ND-1000 spectrophotometer (Thermo Fisher Scientific, MA, USA) and agarose gel electrophoresis, respectively.

PCR amplification of the V3–V4 region of the bacterial 16S rRNA genes was performed using the forward primer 338F (5'-ACTCCTACGGGAGGCAGCA-3') and the reverse primer 806R (5'-GGACTACHVGGGTWTCTAAT-3'). The 16S rRNA gene amplicon sequencing was performed as described previously (Huang et al., 2019).

Sequence data analyses were mainly performed using QIIME2 (Quantitative Insights Into Microbial Ecology, v1.8.0, http://qiime.org/) and R software. The difference between groups of β diversity index was analyzed using R software and presented as a PCA plot. The taxonomic composition analysis at the level of phylum, class, order, family, genus, and species was carried out by QIIME2. The circos plot was conducted online (http://mkweb.bcgsc.ca/tableviewer/). Linear discriminant analysis effect size (LEfSe) was performed to detect differentially abundant taxa across groups using the default parameters (Segata et al., 2011).

The Spearman correlations of microbial taxa with the level of plasma inflammatory cytokine IL-1β were calculated by R software and presented as heat maps.

### 2.9. Cell culture

The human monocytic THP-1 and the normal human colonic epithelial NCM460 cell lines were routinely cultured in RPMI 1640 containing 10% fetal bovine serum, 100 U/ml penicillin, and 0.1 mg/ml streptomycin.

THP-1 was differentiated into M0 macrophages by 100 ng/ml PMA for 24 h. Then, THP-1-derived macrophages in the Con group were maintained in RPMI 1640 without PMA and LPS for 48 h, while THP-1-derived macrophages in the LPS and LPS+Pp group were stimulated with 800 ng/ml LPS. *P. pentosaceus* (Multiplicity of Infection, MOI = 50) was co-cultured with THP-1-derived macrophages in the LPS+Pp group at the same time. The cell supernatant of the LPS+Pp and LPS group was collected after 48 h, respectively. Stimulated THP-1-derived macrophages were collected for the polarization test.

NCM460 cells were stimulated with 1% DSS and co-cultured with supernatants from THP-1-derived macrophages treated with LPS and Pp (the DSS+PS group) or LPS alone (the DSS+LS group). In addition, 10 ng/ml IL-1β was added to the DSS+IL-1β group. These NCM460 cells were collected for apoptosis test and ROS test.

### 2.10. Flow cytometry

The surface markers of cells were detected by flow cytometry. THP-1-derived macrophages were collected after being stimulated. The antibodies used were as follows: FITC anti-human CD86 (BU63) and PE anti-human CD206 (15-2) from BD Biosciences. The apoptosis of NCM460 cells was also detected by flow cytometry. Stimulated NCM460 cells were collected and stained with Annexin V-FITC and PI for 10 min and then detected by BD FACSCalibur (BD Biosciences, CA, USA).

### 2.11. GMrepo database analysis

GMrepo (data repository for gut microbiota) is a database of annotated human gut metagenomes (Wu et al., 2020). The relative abundance of *A. muciniphila* in fecal samples of IBD (UC) and non-IBD patients was from the GMrepo database.

### 2.12. Measuring the abundance of *A. muciniphila* in stool samples

The abundance of *A. muciniphila* was measured by qPCR as described in previous research (Katiraei et al., 2020). DNA was extracted from 20 mg stools using the BIOG DNA Stool Kit following the manufacturer's instructions. The primer for the 16S rRNA gene and *A. muciniphila* is listed in Supplementary Table 1.

### 2.13. *Akkermansia muciniphila* (A. muciniphila) culture and proliferation test

*A. muciniphila* DSM22959 was gifted by Professor Qinghua Yu. It was cultured on BHI solid plate for 72 h in an anaerobic workstation at 37°C for two generations. Then, a single colony was picked for extended culture in a 10 ml BHI liquid medium. *A. muciniphila* was centrifuged at 6,000 r/min for 4 min after extended culture and suspended in saline to prepare bacterial suspension with a turbidity of 1 for further experiments.

The supernatant of NCM460 cultured for 24 h was collected and added with or without 2 mM of H_2_O_2._ In addition, NCM460 cells were stimulated with 1% DSS and co-cultured with supernatants from THP-1-derived macrophages treated with LPS and Pp (the DSS+PS group) or LPS alone (the DSS+LS group). Then, the NCM460 cell supernatants from the DSS+PS group and the DSS+LS group were also collected. Next, the aforementioned collected supernatants (0.5 ml) were added into the culture medium of *A. muciniphila* to a final volume of 10 ml, respectively. To detect the growth rate of *A. muciniphila*, the optical density was measured under a microplate reader at 600 nm at 0 h, 6 h, 12 h, 16 h, 20 h, 24 h, 28 h, and 32 h, respectively.

### 2.14. Statistical analysis

All the data were presented as mean ± standard deviation. Student's *t*-test or Welch's *t*-test was used to assess difference between two groups. The *P*-values lower than or equal to 0.05 were considered significant.

## 3. Results

### 3.1. *P. pentosaceus* CECT8330 ameliorates DSS-induced colitis

**Figure 1:**
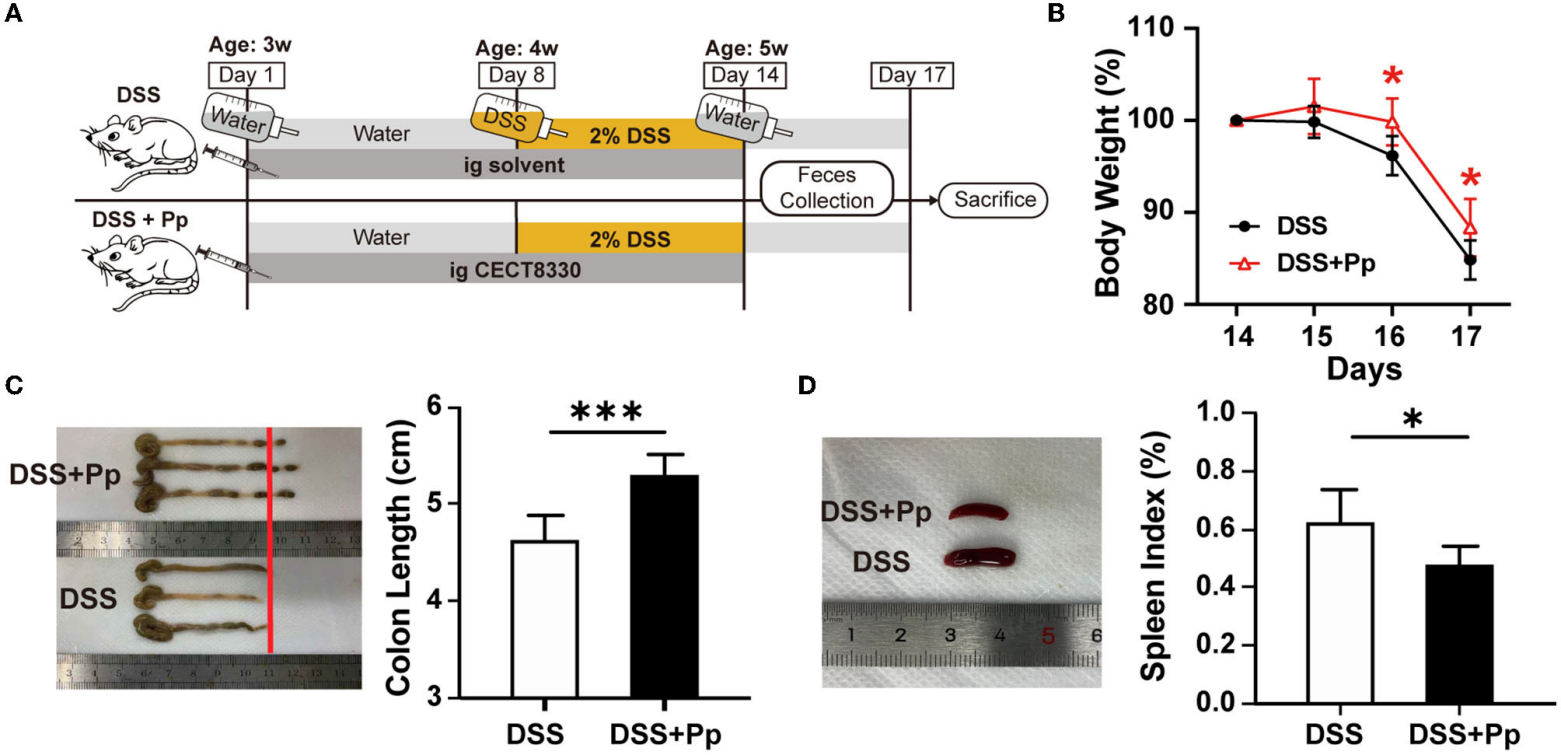
*P. pentosaceus* CECT8330 ameliorates DSS-induced colitis in mice. **(A)** A flow chart showing the animal experiment design. **(B)** The body weight from Day 14 to Day 17; *n* = 6 per group. **(C)** The typical photograph of colons and colon lengths; *n* = 6 per group. **(D)** The typical photographs of spleens and spleen indexes; *n* = 6 per group. Data represent mean ± SD. ^*^P < 0.05, ^***^P < 0.001.

*P. pentosaceus* CECT8330, one of *P. pentosaceus* strains, belongs to the family of *Lactobacillaceae* (Supplementary Figure 1). To assess the effect of *P. pentosaceus* CECT8330 on PIBD, the DSS-induced colitis model was established as reported in a previous study (Bian et al., 2020). 3-week-old juvenile mice were administered with *P. pentosaceus* CECT8330 or solvent by gavage for 14 days (Figure 1A), and 2% DSS was added to the drinking water from the age of 4 weeks. The intake of *P. pentosaceus* CECT8330 significantly reduced the decline of body weight (Figure 1B) and ameliorated DSS-induced features of colitis, including longer colon lengths and smaller spleens (Figures 1C, D), which means *P. pentosaceus* CECT8330 reduces colonic inflammation.

### 3.2. *P. pentosaceus* CECT8330 protects against DSS-induced colonic epithelial injury

**Figure 2:**
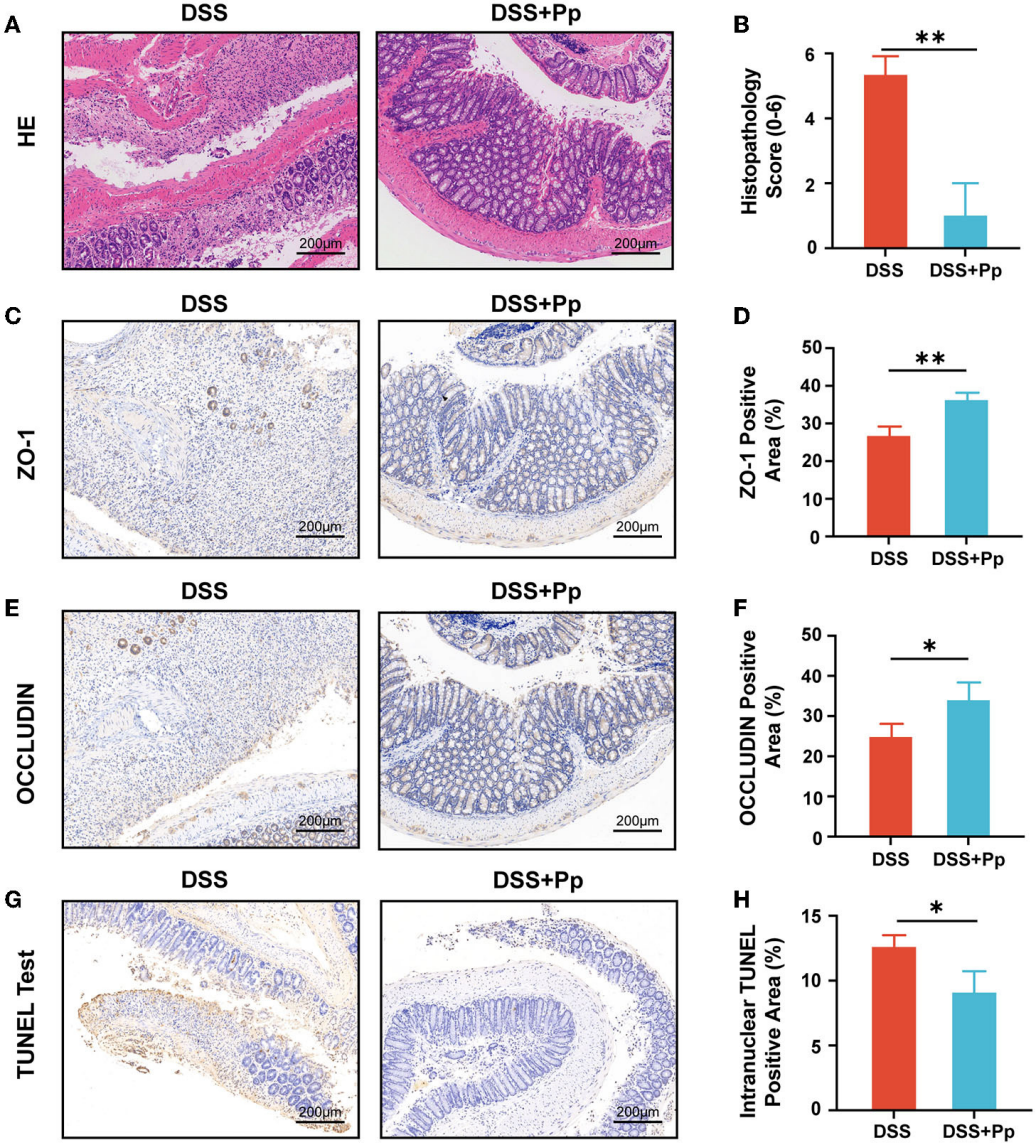
*P. pentosaceus* CECT8330 ameliorates DSS-induced colonic epithelial injury and inflammation in mice. **(A)** Representative images of H&E staining. **(B)** Histopathology score; *n* = 3 per group. **(C)** Representative images of ZO-1 immunohistochemistry staining. **(D)** The percentages of ZO-1 positive area; *n* = 3 per group. **(E)** Representative images of OCCLUDIN immunohistochemistry staining. **(F)** The percentages of OCLLUDIN positive area; *n* = 3 per group. **(G)** Representative images of TUNEL test of mice colon tissue. **(H)** The percentages of intranuclear TUNEL positive area; *n* = 3 per group. Data represent mean ± SD. ^*^P < 0.05, ^**^P < 0.01.

DSS-induced injury of intestinal epithelial cells leads to bacteria and their products disseminating into deeper tissue like submucosa, which thus results in inflammatory responses. The typical histological changes in acute DSS-induced colitis include loss of crypts, epithelial erosion, ulceration, mucin and goblet cell depletion, and granulocyte infiltration in lamina propria and submucosa (Chassaing et al., 2014; Kiesler et al., 2015). As shown in Figures 2A, B, the histological analysis of the large intestine from the DSS group mice showed an obvious loss of goblet cells and crypts, thinner mucus layer, and serious granulocyte infiltration in lamina propria and submucosa, indicating the colonic epithelial injury caused by DSS (the DSS group), whereas *P. pentosaceus* CECT8330 treatment (the DSS + Pp group) could substantially relieve damage of mucosa structure.

Furthermore, we investigated the effect of *P. pentosaceus* CECT8330 on epithelial barrier function by testing the expression levels of tight junction proteins ZO-1 and OCCLUDIN in colonic tissue. Both ZO-1 and OCCLUDIN were upregulated in the colon tissue of mice after the administration of *P. pentosaceus* CECT8330 (Figures 2C–F), and *Zo1* and *Ocln* mRNA levels showed consistent results (Supplementary Figures 2A, B). Meanwhile, *P. pentosaceus* CECT8330 could remarkably relieve DSS-induced apoptosis of intestinal epithelium (Figures 2G, H).

Together, the above results indicate that *P. pentosaceus* CECT8330 ameliorates colitis by improving epithelial barrier function and reducing colonic epithelial injury.

### 3.3. *P. pentosaceus* CECT8330 suppresses colonic epithelial apoptosis by inhibiting NF-κB activation

**Figure 3:**
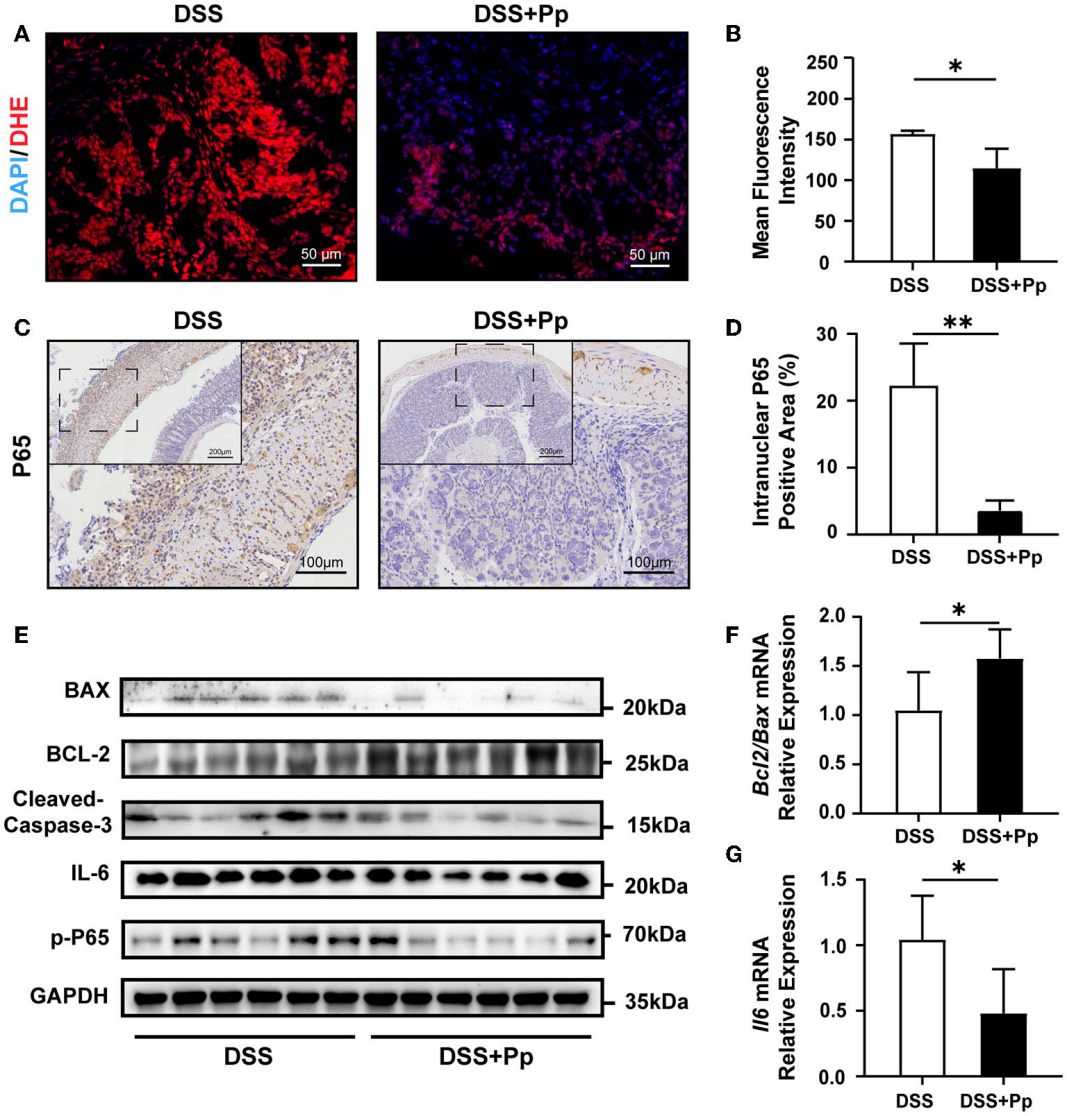
CECT8330 ameliorates colonic epithelial apoptosis and ROS generation in mice by suppressing the NF-κB pathway. **(A)** Representative images of ROS fluorescence of the colon tissue. **(B)** The mean intensity of ROS fluorescence; *n* = 3 per group. **(C)** Representative images of NF-κB P65 immunohistochemistry staining. **(D)** The percentages of intranuclear NF-κB P65 positive area; *n* = 3 per group. **(E)** Western blot analysis for BAX, BCL-2, Cleaved Caspase-3, IL-6, and p-P65 in colon tissue; *n* = 6 per group. **(F)** Relative mRNA expression levels of *Bcl2*/*Bax*; *n* = 5 per group. **(G)** Relative mRNA expression levels of *Il6*; *n* = 5 per group. Data represent mean ± SD. ^*^P < 0.05, ^**^P < 0.01.

Reactive oxygen species (ROS), the main molecules produced during oxidative stress, are highly related to cell apoptosis regulation (D'Autreaux and Toledano, 2007). Thus, we performed a DHE probe fluorescence assay to access the ROS levels and found that *P. pentosaceus* CECT8330 administration decreased the levels of ROS in mice colon tissue (Figures 3A, B). The activation of nuclear factor κB (NF-κB) is highly involved in the inflammatory response in IBD (Schreiber et al., 1998; Andresen et al., 2005). Consistently, the levels of intranuclear NF-κB P65 were decreased in the colon tissue of *P. pentosaceus* CECT8330-treated mice, compared to the DSS control group (Figures 3C, D). NF-κB could activate the transcription of apoptosis-related genes under stress (Campbell et al., 2004), and we further investigated the changes in the levels of apoptosis-related proteins after *P. pentosaceus* CECT8330 treatment. Compared with the DSS group, *P. pentosaceus* CECT8330 treatment remarkably reduced levels of apoptosis-associated proteins BAX and Cleaved Caspase-3, whereas the anti-apoptosis protein BCL-2 was upregulated (Figure 3E, Supplementary Figure 2C); *Bcl2*/*Bax* mRNA levels showed consistent results (Figure 3F). Meanwhile, the protein levels of IL-6 and phospho-p65 (p-P65) were also reduced by *P. pentosaceus* CECT8330 (Figure 3E, Supplementary Figure 2C); and *Il6* mRNA relative expressions were also inhibited by *P. pentosaceus* CECT8330 (Figure 3G). These results suggest that *P. pentosaceus* CECT8330 suppresses DSS-induced enterocyte apoptosis and colitis by inhibiting the NF-κB pathway.

### 3.4. *P. pentosaceus* CECT8330 polarizes macrophages toward the M2 phenotype

Previous studies have shown that the dysfunction of intestinal macrophages leads to the imbalance of intestinal homeostasis (de Souza and Fiocchi, 2016). The increase in pro-inflammatory M1 macrophages detected in the colon of IBD patients and animal models was associated with disease activity (Lissner et al., 2015).

**Figure 4:**
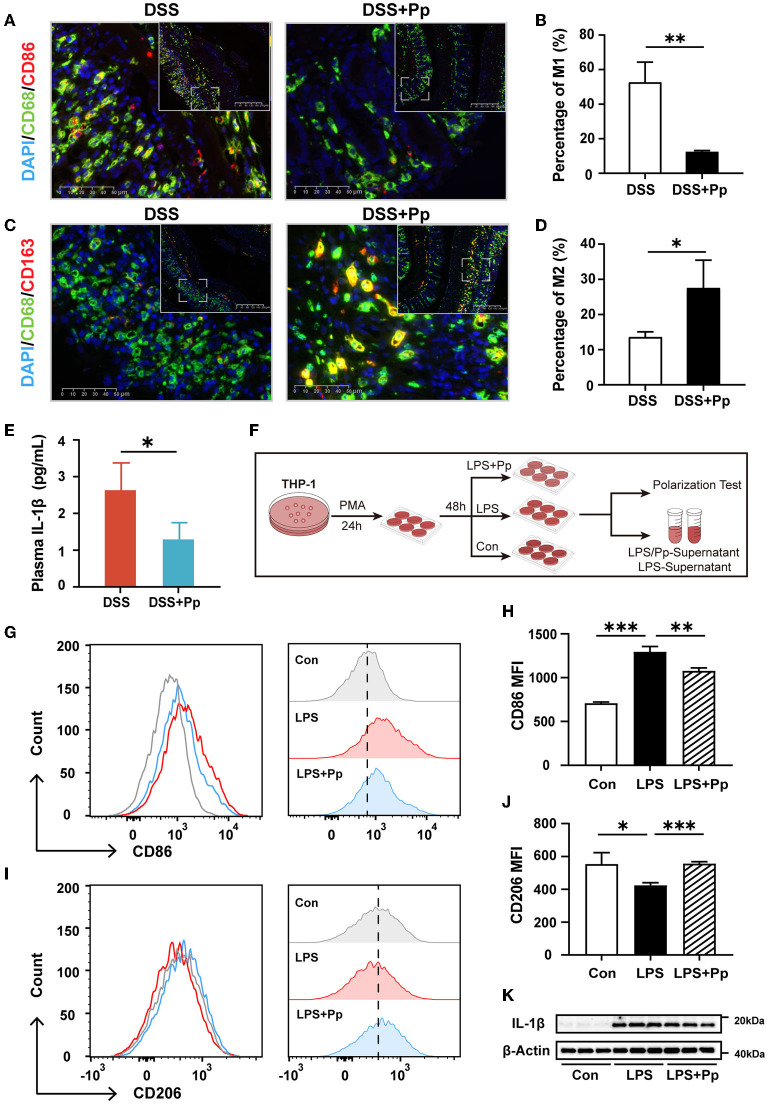
*P. pentosaceus* CECT8330 affects the polarization of macrophages *in vivo* and *in vitro*. **(A)** Representative images of M1 macrophage (CD68^+^ and CD86^+^) immunofluorescence staining of mice colonic tissue. **(B)** The percentages of M1 macrophages (CD68^+^ and CD86^+^) in total macrophages (CD68^+^); *n* = 3 per group. **(C)** Representative images of M2 macrophage (CD68^+^ and CD163^+^) immunofluorescence staining of mice colonic tissue. **(D)** The percentages of M2 macrophages (CD68^+^ and CD163^+^) in total macrophages (CD68^+^); *n* = 3 per group. **(E)** The plasma levels of IL-1β in DSS (*n* = 4) and DSS+Pp (*n* = 3) groups. **(F)** A flow chart depicting the cell experiments about macrophage cell line THP-1. **(G)** Flow cytometry analysis was used for assessing THP-1-derived macrophage polarization under indicated treatment. The histogram of CD86-FITC^+^ cells (M1 macrophages). **(H)** The MFI (Median) of M1 (CD86^+^) macrophages; *n* = 3 per group. **(I)** The histogram of CD206-PE^+^ cells (M2 macrophages). **(J)** The MFI (median) of M2 (CD206^+^) macrophages; *n* = 3 per group. **(K)** Western blot analysis of protein levels of IL-1β in THP-1-derived macrophages with indicated treatment; *n* = 3 per group. Data represent mean ± SD. ^*^P < 0.05, ^**^P < 0.01, ***P < 0.001.

To explore whether macrophage polarization is involved in the protective effect of *P. pentosaceus* CECT8330 on DSS-induced colitis, immunofluorescence staining of M1 and M2 polarization-specific markers was conducted in the mice colon tissue. As shown in Figures 4A–D, *P. pentosaceus* CECT8330 intake remarkably reduced the expression of M1 polarization-specific marker CD86 in the CD68-positive macrophages, while the expression of M2 polarization-specific marker CD163 was increased, suggesting that *P. pentosaceus* CECT8330 blocks macrophages polarizing to M1 but promotes them polarizing to M2. As the pro-inflammatory M1 macrophages decreased, the plasma level of IL-1β in mice treated with *P. pentosaceus* CECT8330 was also reduced (Figure 4E). These results were further corroborated in the *in vitro* THP-1-derived macrophages model (Figure 4F), which showed that *P. pentosaceus* CECT8330 administration inhibited LPS-induced polarization of THP-1-derived macrophages to M1 phenotype (Figures 4G, H) and a concomitant increase in M2 polarization (Figures 4I, J). Additionally, the expression of IL-1β was decreased in THP-1-derivived macrophages treated with *P. pentosaceus* CECT8330, in comparison with LPS stimulation alone (Figure 4K, Supplementary Figure 2D). Both the *in vivo* and *in vitro* results indicate that *P. pentosaceus* CECT8330 suppresses the macrophages polarizing toward the M1 phenotype, leading to decreased secretion of IL-1β.

### 3.5. *P. pentosaceus* CECT8330 inhibits inflammation-induced apoptosis of colonic epithelial cells

**Figure 5:**
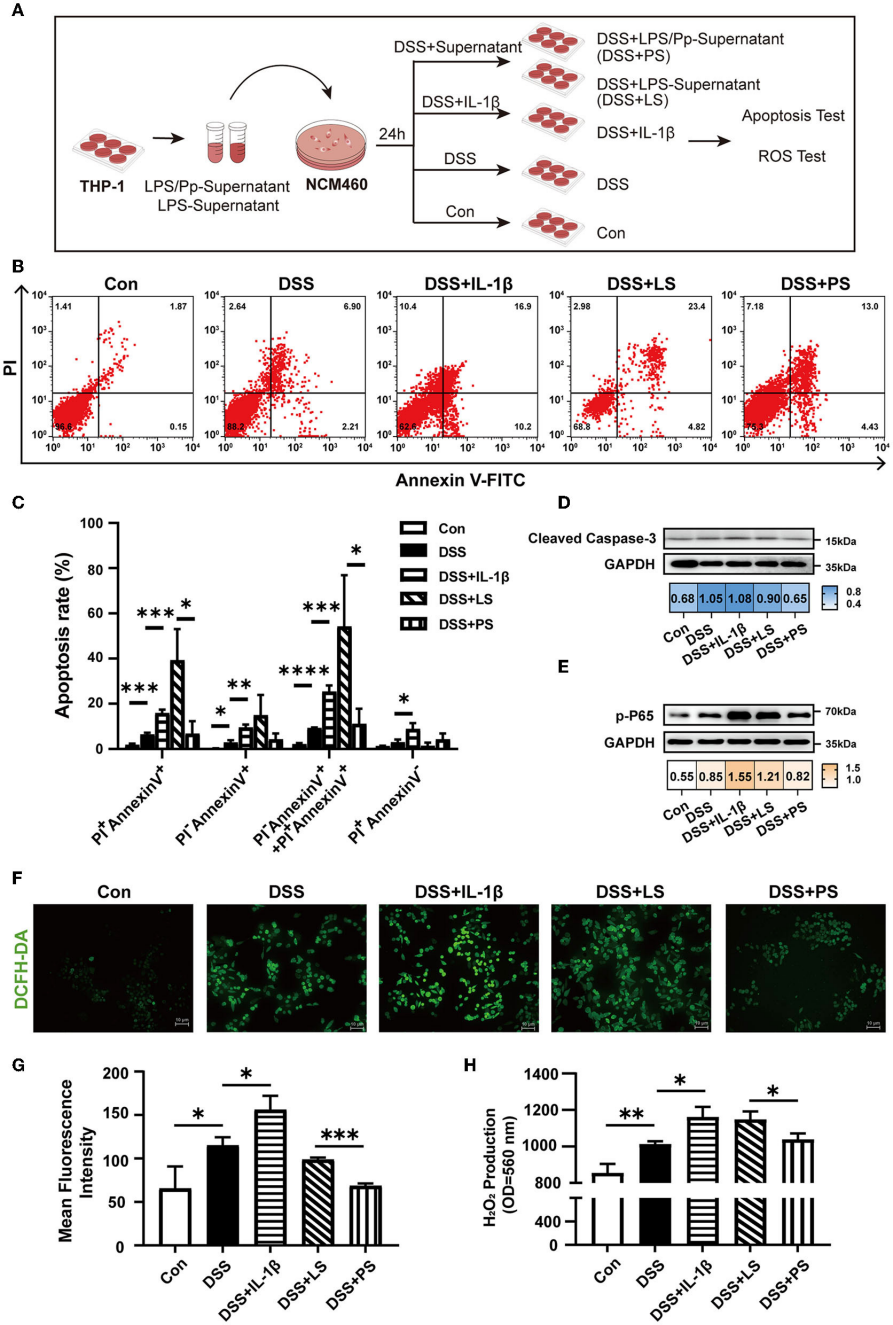
*P. pentosaceus* CECT8330 affects the interaction between macrophages and epithelium. **(A)** A flow chart depicting the cell experiments using human colon epithelial cell line NCM460. **(B)** Flow cytometry analysis was performed to assess apoptosis in NCM460 cells under indicated treatment. **(C)** The percentages of apoptotic cells of late (PI^+^-Annexin V^+^) stages, early (PI^−^-Annexin V^+^) stages, and dead cells (PI^+^-Annexin V^−^); *n* = 3 per group. **(D)** Western blot analysis of protein levels of Cleaved Caspase-3 in NCM460 cells. **(E)** Western blot analysis of protein levels of p-P65 in NCM460 cells. **(F)** Representative images of ROS fluorescence in different groups. **(G)** Mean intensity of ROS fluorescence; *n* = 3 per group. **(H)** H_2_O_2_ production from NCM460 cells. Data represent mean ± SD. *P < 0.05, **P < 0.01, ***P < 0.001, ****P < 0.0001.

We proceeded to investigate whether *P. pentosaceus* CECT8330 inhibits colonic epithelial apoptosis *via* alleviating macrophage-mediated inflammation. Since IL-1β is one of the most important pro-inflammatory cytokines produced by M1 macrophages, and *P. pentosaceus* CECT8330 could suppress M1 polarization and reduce IL-1β production, we further investigated the effects of the supernatants of THP-1-derived macrophages treated with or without *P. pentosaceus* CECT8330 on apoptosis of NCM460 cells, using IL-1β recombinant protein as positive control (Figure 5A). As shown in Figures 5B, C, compared to the control group (DSS + LS), the supernatants of macrophages treated with *P. pentosaceus* CECT8330 (the DSS + PS group) could significantly suppress apoptosis of NCM460 cells. Meanwhile, the levels of Cleaved Caspase-3 and phosphorylated P65 were also decreased in NCM460 cells from the DSS+PS group (Figures 5D, E).

We assumed that IL-1β in the supernatants of THP-1-derived M1 macrophages induce ROS production which further elicits NF-κB activation and apoptosis of NCM460. Therefore, we detected ROS levels in the NCM460 cells after incubation with supernatants from LPS-induced macrophages with or without *P. pentosaceus* CECT8330 treatment (Figure 5A). As shown in Figures 5F–H, in comparison with the DSS+LS group, *P. pentosaceus* CECT8330-treated macrophage supernatants (DSS+PS) could reduce intracellular ROS production, as well as the extracellular production of H_2_O_2_.

Collectively, the aforementioned results suggest that *P. pentosaceus* CECT8330 inhibits apoptosis of colonic epithelial cells *via* suppressing macrophage inflammation.

### 3.6. *P. pentosaceus* CECT8330 increases gut microbiome diversity in mice with DSS-induced colitis

**Figure 6:**
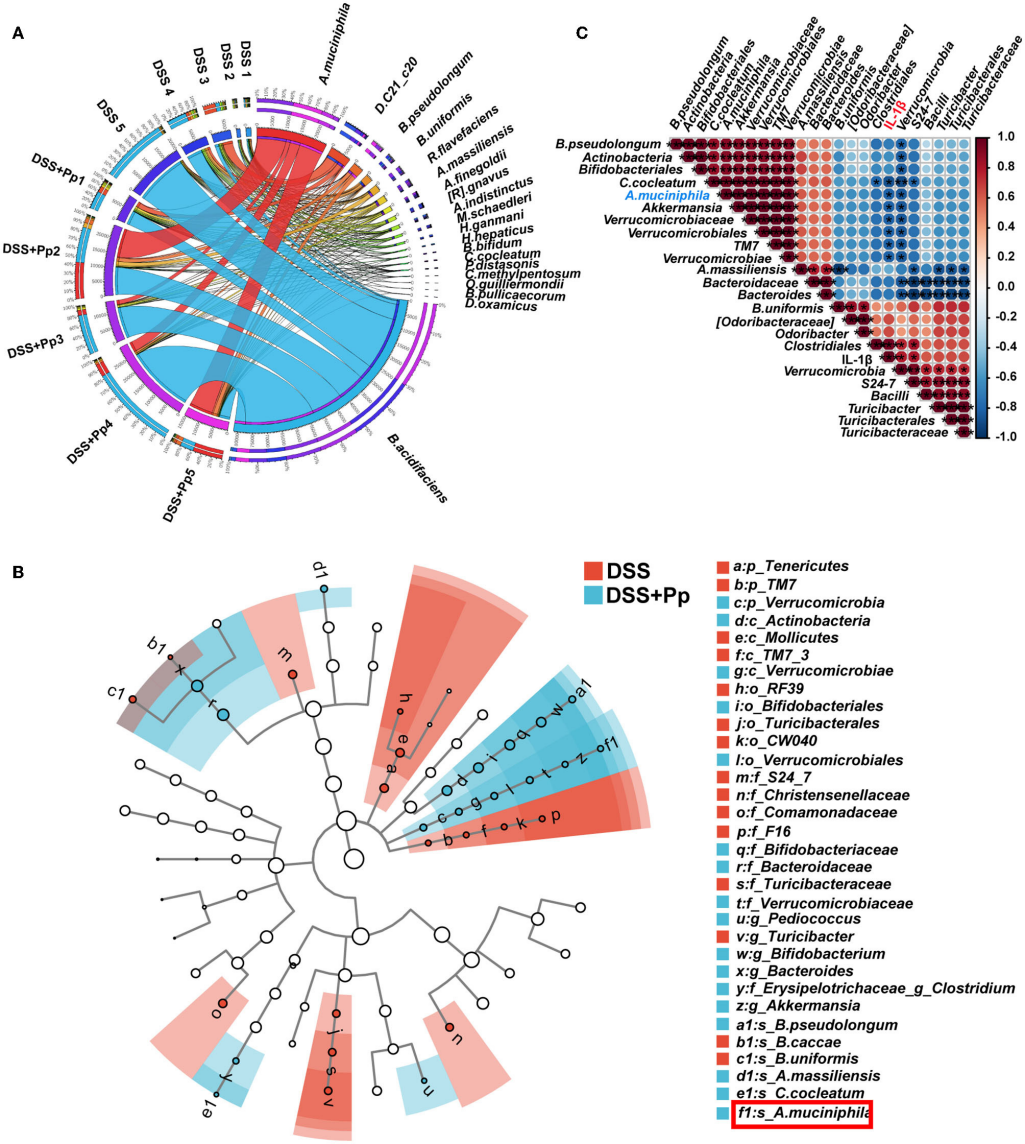
The *A. muciniphila* relative abundance is related to the plasma inflammatory factor IL-1β in mice. **(A)** Circos graph was made to show the top 20 relative abundances of microbial taxa in each sample. *A. muciniphila, Akkermansia muciniphila*; *D. C21_c20, Desulfoviobrio C21_c20*; *B. pseudolongum, Bifidobacterium pseudolongum*; *B. uniformis, Bacteroides uniformis*; *R. flavefaciens, Ruminococcus flavefaciens*; *A. massiliensis, Alistipes massiliensis*; *A. finegoldii, Alistipes finegoldii*; *[R]*. *gnavus, [Ruminococcus] gnavus*; *A. indistinctus, Alistipes indistinctus*; *M. schaedleri, Mucispirillum schaedleri*; *H. ganmani, Helicobacter ganmani*; *H. hepaticus, Helicobacter hepaticus*; *B. bifidum, Bifidobacterium bifidum*; *C. cocleatum, Clostridium cocleatum*; *P. distasonis, Parabacteroides distasonis*; *C. methylpentosum, Clostridium methylpentosum*; *O. guilliermondii, Oscillospira guilliermondii*; *B. pullicaecorum, Butyricicoccus pullicaecorum*; *D. oxamicus, Desulfovibrio oxamicus*; *n* = 5 per group. **(B)** Cladograms of taxonomic hierarchical distribution of biomarkers in DSS and DSS + Pp group; *n* = 5 per group. *A. muciniphila* was labeled by the red box. **(C)** The Spearman correlations of representative microbial taxa and plasma inflammatory cytokine IL-1β between DSS and DSS + Pp group. *P < 0.05, **P < 0.01, ***P < 0.001.

**Figure 7:**
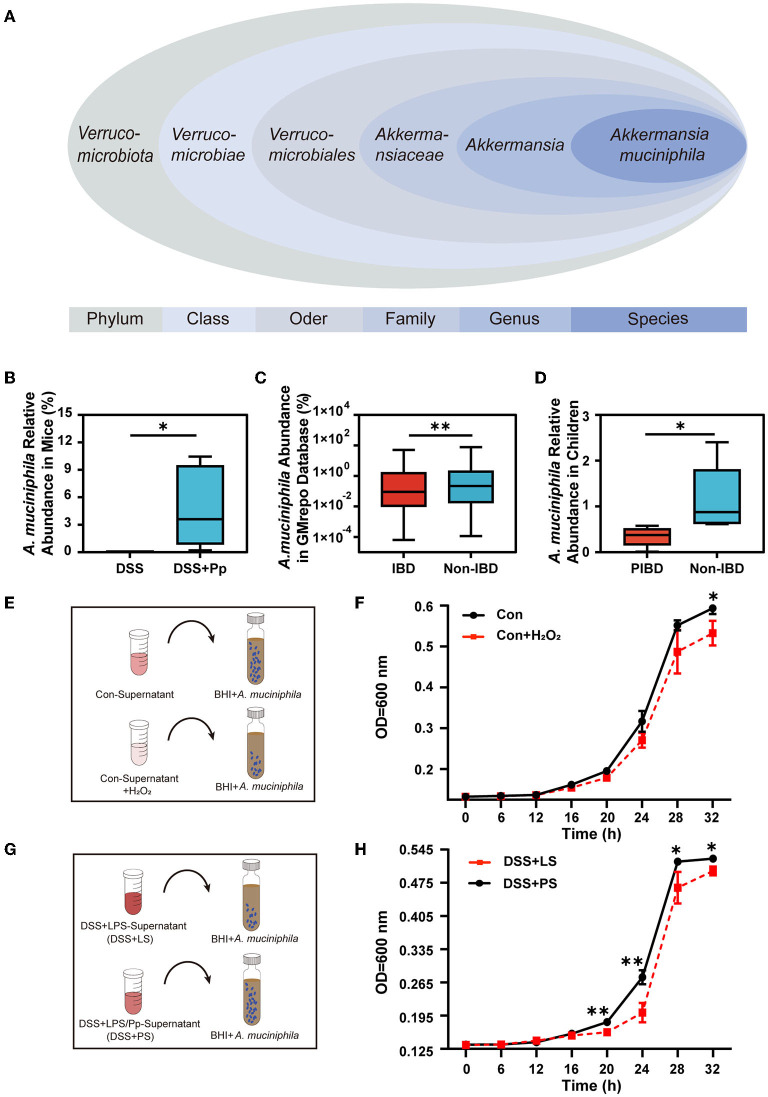
*P. pentosaceus* CECT8330 increases the relative abundance of *A. muciniphila* by regulating ROS generation. **(A)** The classification of *A. muciniphila* according to the NCBI Taxonomy Database. **(B)** Relative abundance of *A. muciniphila* in mice fecal samples between DSS and DSS+Pp groups; *n* = 5 per group. **(C)** Comparison of the *A. muciniphila* abundance in IBD (UC) and non-IBD patients in the GMrepo database. **(D)** Comparison of the abundance of *A. muciniphila* in fecal samples of IBD children and non-IBD children; *n* = 5 per group. **(E)** A diagram depicting the *A. muciniphila* proliferation test. The Con-Supernatant referred to the cell supernatant of NCM460 cultured for 24 h without any interventions, and the Con-Supernatant+H_2_O_2_ group referred to Con-Supernatant with the addition of H_2_O_2_ (2mM). **(F)** The absorbance of *A. muciniphila* medium after being added with supernatants of NCM460 cells with or without H_2_O_2_. **(G)** A diagram depicting the *A. muciniphila* proliferation test. NCM460 cells were stimulated with DSS and co-cultured with supernatants from THP-1-derived macrophages treated with LPS and Pp (DSS+PS group) or LPS alone (DSS+LS group). Supernatants of NCM 460 cells with indicated treatment were then added into the culture medium of *A. muciniphila*. **(H)** The absorbance of *A. muciniphila* medium after added with the supernatants of NCM460 cells. ^*^P < 0.05, ^**^P < 0.01.

It is well-recognized that gut microbes play an essential role in IBD (Ni et al., 2017). Probiotics play a therapeutic role in multiple ways, particularly through interacting with resident microbiota and modulating its function (Sanders et al., 2019). To further investigate how gut microbiota is influenced by *P. pentosaceus* CECT8330, we conducted a 16S rRNA sequence analysis to explore the microbial diversity in fecal samples of DSS-induced colitis mice pretreated with or without *P. pentosaceus* CECT8330 (DSS and DSS+Pp groups). The difference in β diversity index between the two groups was conducted and visualized by a principal component analysis (PCA), and a distinct shift of gut microbiota composition after *P. pentosaceus* CECT8330 administration in colitis mice was revealed (Supplementary Figure 3A). The Venn plot showed 1,868 common OTUs between the two groups (Supplementary Figure 3B). The circos plot showed the relative abundance of the top 20 species in each fecal sample (Figure 6A). Among them, the relative abundance of anaerobic bacteria significantly increased after administration of *P. pentosaceus* CECT8330 (the DSS+Pp group), such as *A. muciniphila, Alistipes massiliensis* (*A. massiliensis*), *Clostridium cocleatum* (*C. cocleatum*), and *Bifidobacterium pseudolongum* (*B. pseudolongum*) (Figure 7B and Supplementary Figure 4A). The enrichment of several taxa, especially *A. muciniphila*, was observed in the DSS+Pp group. Although the relative abundance of *Bacteroides uniformis* (*B. uniformis*) was the largest, there was no difference between the two groups. To find the biomarkers of all classification levels, LEfSe analysis was performed. A cladogram was conducted to show the taxonomic hierarchical distribution of biomarkers in each group of samples (Figure 6B). The histogram of the LDA value distribution of biomarkers was used to show the significantly enriched taxa in each group (Supplementary Figure 4B). Then, to further explore how *P. pentosaceus* CECT8330 exerts positive effects *via* modulating the composition of the gut microbiome, we conducted a correlation analysis between the plasma levels of pro-inflammatory cytokine IL-1β and the relative abundance of gut microbiota, which showed that the level of IL-1β in mice plasma was negatively correlated with the relative abundance of *A. muciniphila, Akkermansia*, and *Verrucomicrobiaceae* (Figure 6C). The aforementioned results suggest that there are several possible differential biomarkers among samples of DSS-induced colitis mice pretreated with or without *P. pentosaceus* CECT8330, such as *A. muciniphila*.

### 3.7. *P. pentosaceus* CECT8330 modifies gut microbiota by affecting *A. muciniphila*

*A. muciniphila*, belonging to the *Verrucomicrobia* (Figure 7A), is considered a prospective probiotic (Zhang et al., 2019). Our results showed that the abundance of *A. muciniphila* in the feces of mice was significantly increased after *P. pentosaceus* CECT8330 administration (Figure 7B). We assumed that *P. pentosaceus* CECT8330 could directly regulate the abundance of *A. muciniphila* to relieve the systemic inflammatory levels. To further investigate the contribution of *A. muciniphila* to IBD, we evaluated the abundance of *A. muciniphila* in IBD and non-IBD patients using the GMrepo database (Figure 7C). We found that the relative abundance of *A. muciniphila* obviously decreased in IBD patients. To confirm this result, we collected the feces of IBD and non-IBD children to detect the abundance of *A. muciniphila* by qPCR (Katiraei et al., 2020) and found that *A. muciniphila* abundance decreased in PIBD patients (Figure 7D).

We next investigated whether increased ROS production in colonic epithelial cells affects the abundance of *A. muciniphila*. After confirming the inhibitory effect of H_2_O_2_ on the proliferation of *A. muciniphila* (Figures 7E, F), we collected the supernatants of NCM460 cells treated with DSS+PS or DSS+LS and added them into the culture system of *A. muciniphila* (Figure 7G). Expectedly, *A. muciniphila* in the DSS+PS treatment group had a higher proliferation capacity than that of the DSS+LS group (Figure 7H).

## 4. Discussion

Genetics, environmental factors, and the host immune system participate in the development of IBD, and their complex interplay results in aberrant immune responses and chronic intestinal inflammation (Knox et al., 2019; Lee and Chang, 2021). The mice about 3 weeks old belong to juvenile mice (Ettreiki et al., 2019; Gorberg et al., 2021). Therefore, we used 3-week-old juvenile mice to simulate the development of IBD in children in our study. *P. pentosaceus* CECT8330 showed a significant amelioration of DSS-induced colitis phenotype in the 3-week-old juvenile mice, including improvements in body weight loss, colon length shortening, pathological structure destruction, and intestinal barrier function (Figures 1, 2). It has been reported that due to the changes in intestinal immune status and intestinal flora, the susceptibility to DSS varies among mice of different ages, and young mice tend to be more tolerant to DSS than old mice (Liu et al., 2020). In other disease-related mouse models, mice of different ages are used for studies because age is believed to have an impact on treatment response (Caputi et al., 2017; Gorberg et al., 2021). However, there is still a lack of probiotic studies in juvenile colitis, which supports the necessity of our follow-up research.

NF-κB is a crucial inflammatory signaling pathway involved in the progression of IBD, which can not only mediate cell apoptosis but also regulate intestinal permeability (Atreya et al., 2008; Ibrahim et al., 2020). P65 is the key transcription factor of the classical NF-κB pathway. Active P65 translocates to the nucleus in a heterodimer with P50, where it binds to the promoter of the target genes and transcriptionally regulates gene expression (Kang et al., 2001). Under the co-stimulation of inflammation and other stress signals, NF-κB P65 could inhibit the transcription of anti-apoptotic genes (Campbell et al., 2004). In the present study, a significant decrease in nuclear P65 and apoptosis of epithelial cells was observed under the treatment of *P. pentosaceus* CECT8330, indicating *P. pentosaceus* CECT8330 inhibits intestinal epithelial cell apoptosis by inhibiting the NF-κB signaling pathway (Figure 3).

There is increasing evidence to suggest that macrophages play an important role in regulating the intestinal immune system and inflammatory microenvironment and can be used as drug targets for the treatment of IBD (Leung et al., 2013; Zhang et al., 2020). In our study, after *P. pentosaceus* CECT8330 administration, M1 polarization of macrophages was decreased, M2 polarization was increased, and the inflammatory cytokine IL-1β was decreased significantly both in the juvenile mice model and in *in vitro* cellular model (Figure 4) (Hunter et al., 2010). Pro-inflammatory cytokines, such as IL-1β and IL-6, were involved in the communication between macrophages and epithelial cells in the development of IBD (Tatiya-Aphiradee et al., 2018). Our study proved that additional administration of IL-1β aggravated DSS-induced epithelial cell apoptosis and increased ROS production. After administration of *P. pentosaceus* CECT8330, IL-1β was less produced with the decrease of M1 polarization, and the inflammatory stimulation to epitheliums was weakened, warranting the recovery of the epithelial barrier (Figure 5). These observations suggest that *P. pentosaceus* CECT8330 inhibits apoptosis of epithelial cells by regulating macrophage function.

*A. muciniphila* is a bacterium that can use degraded mucin as an energy source and is generally abundant in healthy microbiota but reduced in the guts of IBD patients (Png et al., 2010; Bian et al., 2019), which is consistent with our results (Figure 7). The oxygen hypothesis assumes that in an inflammatory gut environment, oxygen levels and free oxygen radicals increase (Zhu and Li, 2012; Rigottier-Gois, 2013), leading to further changes in bacterial communities (Rigottier-Gois, 2013). We found that the contents of four anaerobic bacteria (*A. muciniphila, A. massiliensis, C. cocleatum*, and *B. pseudolongum*) in the gut were significantly increased and the level of ROS was decreased after *P. pentosaceus* CECT8330 treatment. Furthermore, decreased ROS contributes to decreased NF-κB activation under *P. pentosaceus* CECT8330 treatment, as ROS are the important inducers of NF-κB (Marino-Merlo et al., 2019). Our results also showed that an increase in ROS could reduce the proliferation of *A. muciniphila*. The change in flora was closely related to the change in the intestinal inflammatory environment (Figure 6). Therefore, *P. pentosaceus* CECT8330 may help reshape the intestinal microbiome and reverse the ecological dysregulation after alleviating intestinal inflammation, particularly promoting the proliferation of beneficial bacteria such as *A. muciniphila*.

At present, several studies have confirmed that *P. pentosaceus* CECT8330 was safe and effective in the treatment of child-related diseases including crying syndrome and functional gastrointestinal disorders clinically (Astó et al., 2021; Chen et al., 2021). According to our current findings, *P. pentosaceus* CECT8330 has the effect of alleviating intestinal inflammation, so it should have the potential for applications in the treatment of PIBD. With the progress of technology, microorganisms are widely used in the field of food science (Chen et al., 2020; Zwirzitz et al., 2022). It was reported that microbial preparations including probiotics can be considered to help alleviate diseases through food supplements (Choi et al., 2022). In the follow-up clinical study, we will try to optimize the intake of probiotics and consider *P. pentosaceus* CECT8330 as a dietary supplement to relieve the symptoms of pediatric IBD.

## 5. Conclusion

Our study demonstrates that *P. pentosaceus* CECT8330 has a significant protective and therapeutic effect on colitis in juvenile mice. It can maintain the integrity of the intestinal barrier and inhibit intestinal epithelial apoptosis not only by inhibiting the NF-κB inflammatory signaling but also by regulating the M1/M2 polarization of macrophages. With the decrease in IL-1β secreted by M1 macrophages, the production of ROS in intestinal epitheliums was reduced, which is conducive to remodeling intestinal flora. In conclusion, our study reveals that *P. pentosaceus* CECT8330 reprograms macrophages from a pro-inflammatory M1 phenotype to an anti-inflammatory M2 phenotype, leading to the alleviation of colitis induced by DSS in juvenile mice. *P. pentosaceus* CECT8330 can be used as a potential probiotic dietary supplement to improve IBD, especially beneficial for pediatric patients.

## Data availability statement

The raw unprocessed gene datasets of 16S rRNA, which were generated during the current study, are available with the NCBI Sequence Read Archive (SRA), accession number PRJNA970935.

## Ethics statement

The studies involving human participants were reviewed and approved by Ruijin Hospital Ethics Committee, Shanghai Jiao Tong University School of Medicine. Written informed consent to participate in this study was provided by the participants' legal guardian/next of kin. The animal study was reviewed and approved by Shanghai Jiao Tong University School of Medicine Institutional Animal Care and Use Committees.

## Author contributions

PL, CX, and YJG designed the project. HH, CP, XXC, XHC, and MJ performed the experiments. HH, CP, YQG, JX, and JH processed the data. HH, CP, and YJG wrote the manuscript. PL and CX revised the manuscript. All authors contributed to the article and approved the submitted version.
