# Supplementary material for: Probiotic lactic acid bacteria alleviate pediatric IBD and remodel gut microbiota by modulating macrophage polarization and suppressing epithelial apoptosis
Source: Front Microbiol. 2023 Jun 15;14:1168924. doi: 10.3389/fmicb.2023.1168924 (PMC10308112; doi:10.3389/fmicb.2023.1168924)
Supplement: Supplementary file 1 [file Data_Sheet_1.docx]

**Supplementary Materials**

**Supplementary Table 1.** Primers for qPCR

|  | Primers |
| --- | --- |
| *Zo1 (tjp1)* | Forward: GGG GCC TAC ACT GAT CAA GA |
|  | Reverse: TGG AGA TGA GGC TTC TGC TT |
| *Ocln* | Forward: ACG GAC CCT GAC CAC TAT GA |
|  | Reverse: TCA GCA GCA GCC ATG TAC TC |
| *Bcl2* | Forward: GCT ACC GTC GTG ACT TCG C |
|  | Reverse: CCC CAC CGA ACT CAA AGA AGG |
| *Bax* | Forward: AGA CAG GGG CCT TTT TGC TAC |
|  | Reverse: AAT TCG CCG GAG ACA CTC G |
| *Il6* | Forward: ACC AGA GGA AAT TTT CAA TAG GC |
|  | Reverse: TGA TGC ACT TGC AGA AAA CA |
| *Gapdh* | Forward: GGC AAA TTC AAC GGC ACA GTC AAG |
|  | Reverse: TCG CTC CTG GAA GAT GGT GAT GG |
| *A. muciniphila* | Forward: CAG CAC GTG AAG GTG GGG AC |
| *16S rRNA* | Reverse: CCT TGC GGT TGG CTT CAG AT  Forward: CAG CAC GTG AAG GTG GGG AC  Reverse: CCT TGC GGT TGG CTT CAG AT |


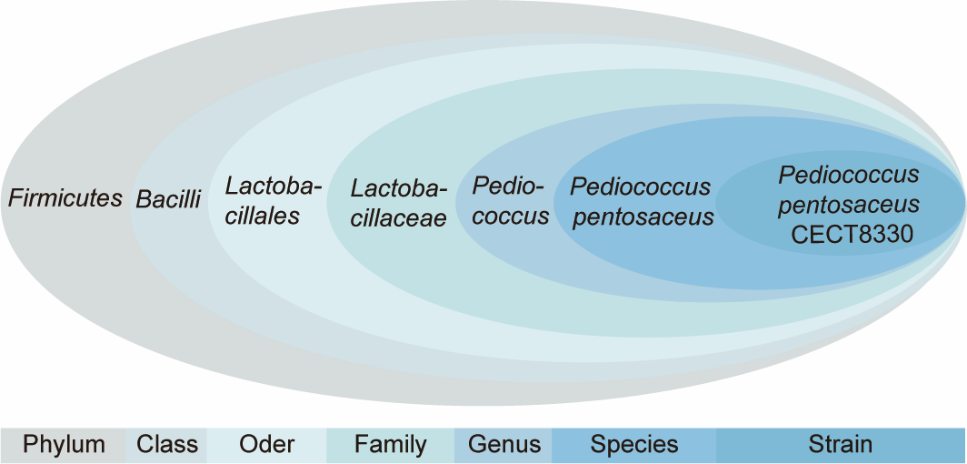


**Supplementary Figure 1. The classification of *P. pentosaceus* CECT8330 according to NCBI Taxonomy Database.**


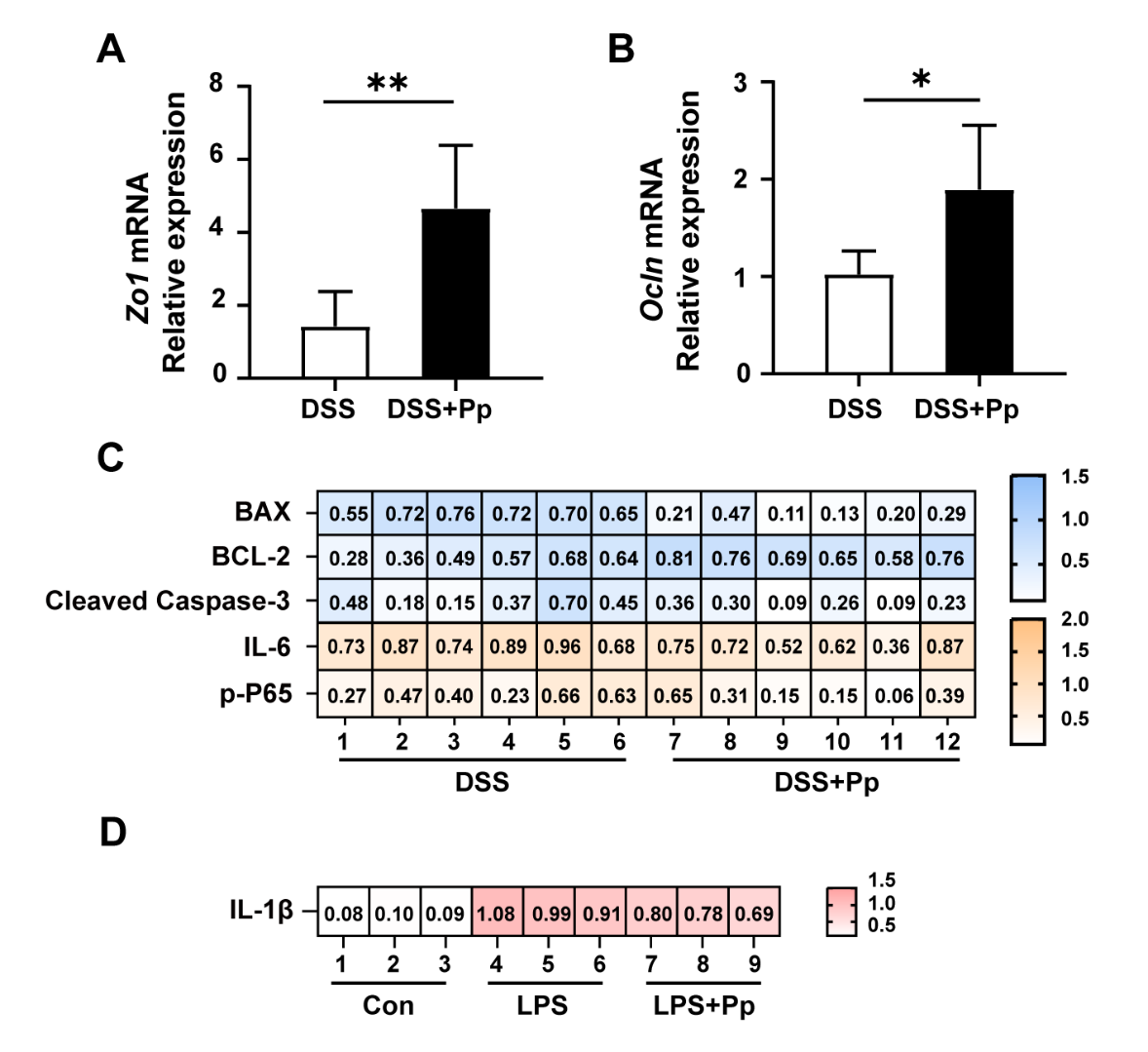


**Supplementary Figure 2. Quantitative analysis of mRNA expressions and protein levels.** (A) The relative expression of *Zo1* mRNA; n=5 per group. (B) The relative expression of *Ocln* mRNA; n=5 per group. (C) The quantitative analysis of target protein/GAPDH in Figure 3E. (D) The quantitative analysis of IL-1β/β-Actin in Figure 4K. Data represent mean ± SD. **P*＜0.05*, **P*＜0.01*.*


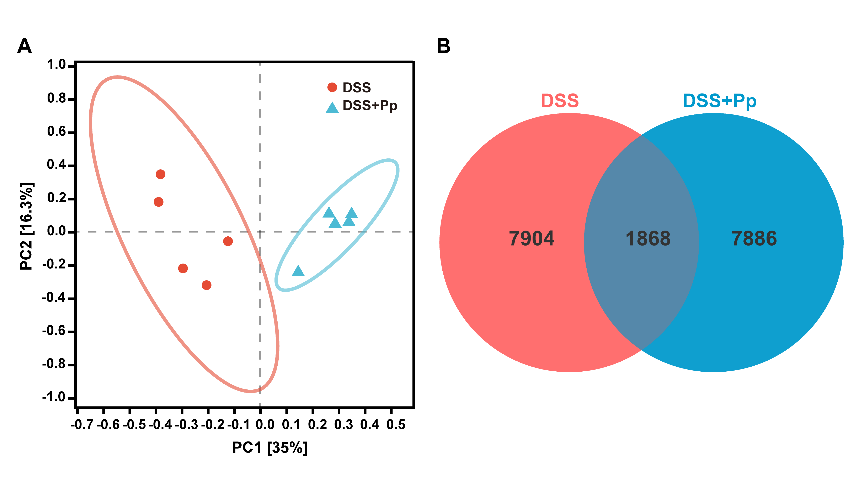


**Supplementary Figure 3. *P. pentosaceus* CECT8330 alters the gut microbiota composition**. (A) The PCA plot indicated the β diversity analysis of the microbiota. Each plot represents a sample. (B) The Venn diagram showed the different numbers of OTUs in two groups.


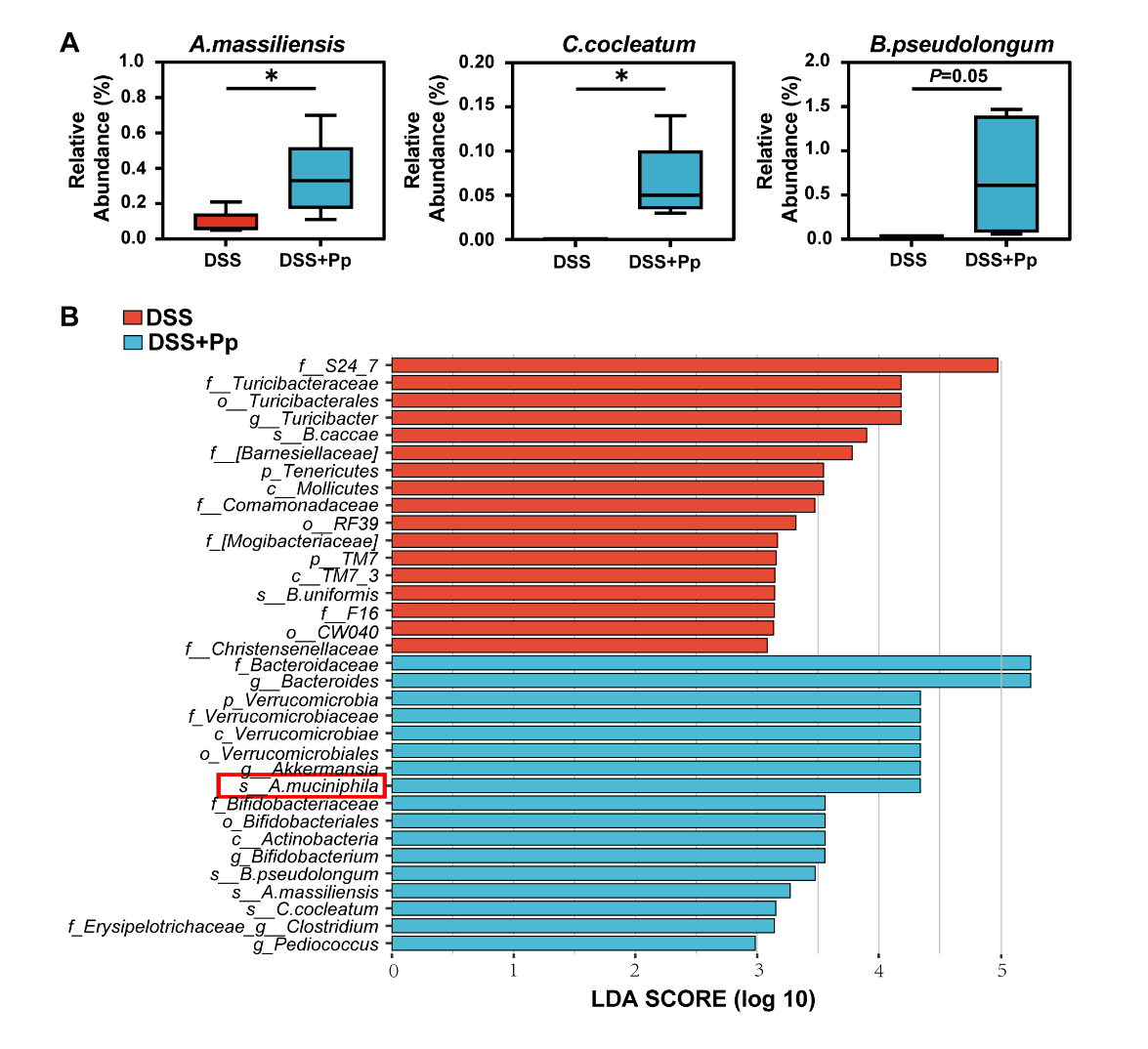


**Supplementary Figure 4. *P. pentosaceus* CECT8330 affects gut microbiota.** (A) Relative abundance of *A. massiliensis*, *C. cocleatum*, *B. pseudolongum* in mice fecal samples between two groups; n=5 per group. (B) Distribution histograms of taxonomic unit with significant difference between DSS and DSS + Pp group; n=5 per group. P, phylum; c, class; o, order; f, family; g, genus; s, species. *A. muciniphila* was labeled by the red box. Data represent mean ± SD. **P*＜0.05.
